# Radiofrequency ablation for peribiliary hepatocellular carcinoma: propensity score matching analysis

**DOI:** 10.1186/s13244-025-01919-5

**Published:** 2025-02-19

**Authors:** Jin Cui, Xinzi Sui, Kaiwen Liu, Min Huang, Yuanwen Zheng, Xinya Zhao, Gongzheng Wang, Ximing Wang

**Affiliations:** 1 Department of Radiology, Shandong Provincial Hospital Affiliated to Shandong First Medical University, Jinan, China; 2 Department of Radiology, Linyi People’s Hospital, Linyi, China; 3 Department of Laboratory, Shandong Provincial Hospital Affiliated to Shandong First Medical University, Jinan, China; 4 Department of Hepatobiliary Surgery, Shandong Provincial Hospital Affiliated to Shandong First Medical University, Jinan, China

**Keywords:** Hepatocellular carcinoma, Peribiliary location, Radiofrequency ablation, Therapeutic outcome

## Abstract

**Objectives:**

At present, there are no established clinical guidelines for radiofrequency ablation (RFA) of peribiliary hepatocellular carcinoma (HCC). Therefore, the aim of this study was to compare the long-term outcomes of RFA for peribiliary vs. non-peribiliary HCC.

**Methods:**

This retrospective study included 282 patients with peribiliary HCC (*n* = 109) or non-peribiliary HCC (*n* = 173) who received RFA between February 2013 and May 2021. Local tumor progression (LTP), overall survival (OS), disease-free survival (DFS), and complications were compared before and after propensity score matching (PSM).

**Results:**

Before PSM, there were no significant differences in 5-year LTP rates (26.3% vs. 23.6%, *p* = 0.602), OS rates (56.6% vs. 68.0%, *p* = 0.586), or DFS rates (22.9% vs. 25.7%, *p* = 0.239) between the peribiliary and non-peribiliary groups. After PSM, there were no significant differences in the 1-, 3-, and 5-year LTP rates (13.0%, 23.1%, and 26.3% vs. 12.1%, 25.1%, and 28.2%, respectively, *p* = 0.857), OS rates (97.2%, 73.5%, and 56.6% vs. 95.3%, 79.5%, and 70.6%, *p* = 0.727), or DFS rates (59.4%, 29.4%, and 22.9% vs. 64.2%, 33.1%, and 23.8%, *p* = 0.568) between the peribiliary non-peribiliary groups. Peribiliary location was not a significant prognostic factor for LTP (*p* = 0.622) or OS (*p* = 0.587). In addition, mild intrahepatic bile duct dilatation was more frequent in the peribiliary group (9.2% vs. 2.8%, *p* = 0.045).

**Conclusion:**

Long-term outcomes of RFA were similar for peribiliary and non-peribiliary HCC. RFA is a viable alternative for treatment of peribiliary HCC.

**Critical relevance statement:**

The local tumor progression (LTP), overall survival (OS), and disease-free survival (DFS) rates after radiofrequency ablation (RFA) were similar for peribiliary and non-peribiliary hepatocellular carcinoma (HCC).

**Key Points:**

- There are currently no clinical guidelines for radiofrequency ablation (RFA) of peribiliary hepatocellular carcinoma (HCC).
- Local tumor progression, overall survival, and disease-free survival after RFA were similar for peribiliary and non-peribiliary HCC.
- RFA is a viable alternative for the treatment of peribiliary HCC.

**Graphical Abstract:**

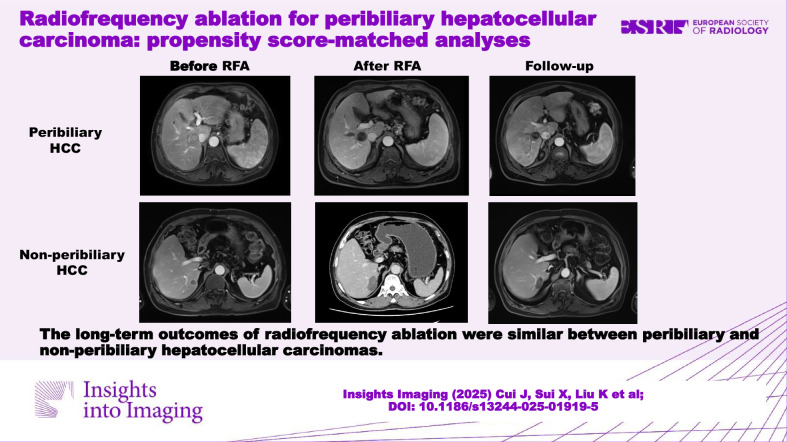

## Introduction

Radiofrequency ablation (RFA) is widely accepted as an effective therapeutic modality for early-stage hepatocellular carcinoma (HCC) [1]. Due to minimal invasiveness, RFA can reduce the duration of surgery and postoperative hospitalization, while maintaining adequate hepatic functional reserve as compared to surgical resection. Unlike surgical resection via laparoscopic or the open approach, RFA is generally performed percutaneously. The location of the tumor is a critical factor influencing the success of RFA, especially for peribiliary HCC [1–5]. Peribiliary HCC is defined as a tumor abutting the first- or second-order branches of the intrahepatic bile duct, with a distance ≤ 5 mm from the bile duct to the tumor margin [6, 7]. The intrahepatic bile duct, which enters the liver through the invagination of Glisson’s capsule and runs along the portal vein and hepatic artery, is not readily visible during RFA procedures. Currently, there are no clinical guidelines for RFA of peribiliary HCC.

Peribiliary location of HCC may increase the difficulty of the RFA technique [8–10]. RFA uses the radiofrequency energy generated from the tip of the electrode to kill tumor cells. However, the heat generated by the electrode may damage the peritumoral bile duct, resulting in biliary complications. The biliary complication rate after RFA is reportedly 1–12%, including bilioma, hemobilia, and biliary stricture [11, 12]. The incidence of hemobilia, which may be treated by embolizing the culprit artery or percutaneous transhepatic biliary drainage, ranges from 0 to 0.3% [12, 13]. RFA of peribiliary HCC may result in incomplete tumor ablation and unfavorable long-term outcomes due to damage to the bile duct or vasculature around the tumor [5, 14].

Although RFA is the first-line treatment option for ≤ 3 cm HCC, in real life, non-negligible part of RFAs are performed in 3–5 cm HCC [15, 16]. Recent advancements in ablation techniques have extended the application of thermal ablation to 3–5 cm HCC, and provide better control of the tumors, thereby improving prognosis [17–20]. RFA has been used as a first-line therapy for medium-sized HCC (3–5 cm) and tumors in challenging locations (i.e., subcapsular and perivascular) to achieve satisfactory outcomes [21–28]. Until recently, only a few studies have investigated the safety and efficacy of RFA for peribiliary HCC [29–31]. The long-term therapeutic outcomes of RFA for peribiliary HCC have not been well characterized until now.

Therefore, the aim of the present study was to compare the long-term outcomes of RFA for peribiliary and non-peribiliary HCC using propensity score matching (PSM). Potential risk factors for local tumor progression (LTP), disease-free survival (DFS), and overall survival (OS) after RFA were also identified. The results of this study provide treatment options for peribiliary HCC as a minimally invasive technique.

## Materials and methods

### Patients

**Fig. 1:**
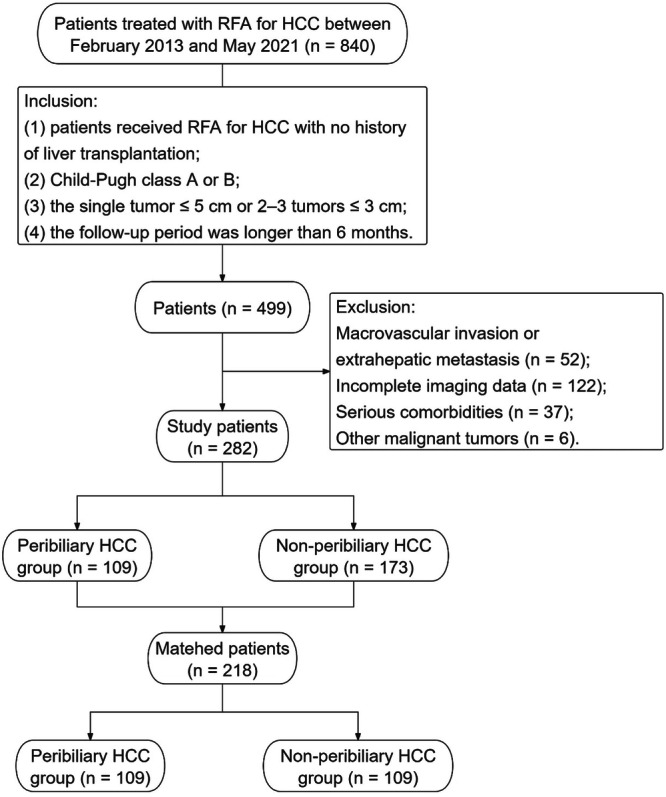
Flow chart for patient selection. HCC, hepatocellular carcinoma; RFA, radiofrequency ablation

The protocol of this retrospective study was approved by the Institutional Review Board of Shandong Provincial Hospital. Due to the retrospective nature of this study, the requirement for informed consent was waived. The medical records of 840 consecutive patients who received RFA for HCC between February 2013 and May 2021 in Shandong Provincial Hospital were retrospectively reviewed. Of these, 499 patients met the following inclusion criteria: (1) patients received RFA for HCC with no history of liver transplantation; (2) Child-Pugh class A or B; (3) HCC within the single tumor ≤ 5 cm or 2–3 tumors ≤ 3 cm; and (4) the follow-up period > 6 months. The exclusion criteria were (1) macrovascular invasion or extrahepatic metastasis on images before initial treatment (*n* = 52); (2) incomplete imaging data (*n* = 122); (3) other serious comorbidities, such as renal failure and congestive heart failure (*n* = 37); and (4) other malignant tumors (*n* = 6). Finally, 282 patients were enrolled in this study. A detailed workflow of the patient enrollment process is shown in Fig. 1.

**Fig. 2:**
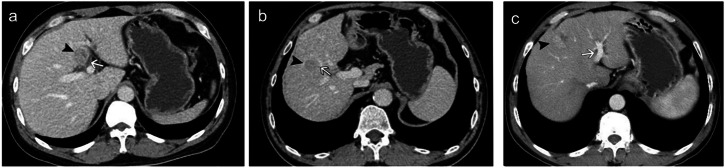
Images of peribiliary HCC (**a**, **b**) and non-peribiliary HCC (**c**). **a** Axial CT image of peribiliary HCC (black arrowhead) adjacent to the first-order branches of the intrahepatic bile duct (white arrow) with distance ≤ 5 mm. **b** Axial CT image of peribiliary HCC (black arrowhead) adjacent to the second-order branches of the intrahepatic bile duct (white arrow) with distance ≤ 5 mm. **c** Axial CT image of non-peribiliary HCC (black arrowhead) distal to the intrahepatic bile duct (white arrow) with distance > 5 mm

HCC was identified in accordance with the practice guidelines of the American Association for the Study of Liver Diseases [2]. Based on multiphase dynamic computer tomography (CT) or magnetic resonance imaging (MRI), the patients were assigned to the peribiliary group (*n* = 109) and the non-peribiliary group (*n* = 173). Peribiliary HCC was defined as a tumor that abutted the first- or second-order branches of the intrahepatic bile duct, with a distance ≤ 5 mm from the bile duct to the tumor margin (Fig. 2) [6, 7]. The first-order bile ducts were the main right and left hepatic ducts, while the second-order bile ducts were the anterior and posterior segment hepatic ducts in the right and left liver lobes [32]. To minimize interpretation variability and potential misclassification, all images of peribiliary and non-peribiliary HCC were independently analyzed by two radiologists with at least 10 years of experience in diagnostic imaging. For cases with disagreement regarding the tumor location between the two radiologists, a final decision was reached through consensus.

Baseline clinical characteristics included age, sex, etiology (hepatitis B virus infection, hepatitis C virus infection, and others), antiviral treatment, serum levels of alpha-fetoprotein (AFP), alanine aminotransferase, and aspartate aminotransferase, model for end-stage liver disease (MELD) score, albumin-bilirubin (ALBI) grade, liver cirrhosis, tumor diameter, tumor number, total ablation time, and tumor location (Couinaud segment). The patients with either esophageal varices or splenomegaly with thrombocytopenia (platelet count ≤ 100 × 10^3^ /μL) were diagnosed as portal hypertension [33]. The total ablation time was defined as the interval from the beginning to the end of RFA.

### Treatment and follow-up

Transcatheter arterial chemoembolization (TACE) was performed by slowly injecting 30–50 mg doxorubicin mixed with 5–10 mL of lipiodol (Guerbet, Villepinte, Seine-Saint-Denis, France) into selected tumor-feeding arteries until cessation of blood flow. The dosage of doxorubicin used for individuals was based on surface area, liver function, and body weight [34–36]. Whether patients received TACE before RFA was determined based on tumor factors, treatment compliance, and economic status. RFA usually followed TACE within 4 weeks.

All RFA procedures were carried out by interventional radiologists with at least 5 years of experience in ablation guided by ultrasound or CT. The RFA therapeutic instrument (Cool-tip RFA; model CTRF220; Covidien LLC) was applied with a power of 0–200 W and a frequency of 480 kHz. Multiple overlapping ablation was used for large index tumors. Whether complete ablation after RFA was achieved was assessed by contrast-enhanced CT or MRI. Complete ablation was defined as a hypo-attenuated area covering the entire tumor without contrast enhancement. For a residual unablated tumor, RFA was repeated to achieve complete tumor necrosis. In our hospital, treatment decisions are made by multidisciplinary teams after a comprehensive assessment of liver function, performance status, patient preference, and tumor burden [37, 38].

All patients were followed up with laboratory tests and ultrasound, CT, or MRI at 1 month after RFA, every 3 months during the first year, and 3–6 months thereafter. Comprehensive diagnostic measures, such as whole-body bone scans and brain MRI, were conducted for suspected extrahepatic recurrence based on clinical symptoms, unexplained elevation of AFP levels, or findings on chest radiographs. For recurrent tumors confirmed during follow-up, therapeutic modalities such as surgical resection, RFA, microwave ablation, TACE, systemic therapies, or other treatment were performed in accordance with the characteristics of tumor recurrence the general condition and liver function of the patient.

### Outcome assessment

The long-term therapeutic outcomes, including LTP, OS, and DFS, were compared between the peribiliary and non-peribiliary groups before and after PSM. LTP was defined as the new appearance of a tumor at the edge of the ablation zone on follow-up images [39]. OS was defined as the time from initial treatment to death or last follow-up (April 24, 2022). DFS was defined as the time from initial treatment to tumor recurrence, death, or last follow-up.

The criteria for complications were based on the definitions of the Society of Interventional Radiology [40]. Major complications were defined as events that required additional therapeutic interventions or prolonged hospitalization, while all other adverse events were classified as minor complications. Severe intrahepatic bile duct dilatation was defined as dilatation that affected two or more hepatic subsegments, while mild intrahepatic bile duct dilatation affected only one hepatic subsegment [8]. Postoperative liver decompensation was defined as the development of at least one of the following signs within 90 days after RFA treatment: ascites, hepatic encephalopathy, variceal bleeding, or jaundice [41–43].

Subgroup analyses of LTP and OS were performed based on the maximum tumor diameter, tumor number, and the branches of peritumoral bile duct (the first- or second-order branches) [32].

### PSM

To minimize the influence of confounding factors and selection bias, PSM was performed with a matching ratio of 1:1 and caliper of 0.1. Variables that were deemed clinically important or significantly different at baseline (*p* < 0.10) were used for PSM. The variables included in the PSM model were age, sex, liver cirrhosis, portal hypertension, antiviral treatment, serum levels of AFP, alanine aminotransferase, and aspartate aminotransferase, MELD score, ALBI grade, Child-Pugh class, maximum tumor diameter, and tumor number. After matching, 218 patients were selected for further analysis. The effect size was reported as a standardized mean difference (SMD) to evaluate the covariate of balance, where SMD < 0.2 indicates a negligible difference [44–46].

### Statistical analysis

All statistical analyses were conducted using IBM SPSS Statistics for Windows (version 26.0; IBM Corporation, Armonk, NY, USA) and R software (version 4.1.3, https://www.r-project.org/). Continuous variables were analyzed using the Mann–Whitney test and are presented as medians and interquartile ranges, while categorical variables were analyzed by Fisher’s exact test or Pearson’s χ2 test, and are presented as numbers and percentages. Cumulative LTP, OS, and DFS curves were plotted using the Kaplan–Meier method and estimated with the log-rank test. Prognostic factors for LTP and OS were identified with univariate and multivariate Cox proportional hazards regression models. Significant clinical factors (*p* < 0.1) identified by univariate Cox analysis were included in the multivariate model. A two-tailed *p*-value < 0.05 was considered statistically significant.

## Results

### Patient characteristics

The baseline characteristics of patients in both the total and PSM cohorts are shown in Table 1. Of the 282 patients included in this study, 109 patients were assigned to the peribiliary group and 173 to the non-peribiliary group. Tumors in the peribiliary group were not abutting the common bile duct. Patients in the peribiliary group had larger maximum tumor diameters and more instances of multiple tumors than those in the non-peribiliary group (both, *p* < 0.05). In addition, the prevalence of Child-Pugh class B was greater in the non-peribiliary group than in the peribiliary group (*p* = 0.040). There were no significant differences in any other variables between the two groups. After PSM, the characteristics of 109 pairs of matched patients (i.e., tumor diameter, tumor number, Child-Pugh class, and TACE before RFA) were well balanced (all, *p* > 0.05).

**Table 1:** Baseline characteristics of study patients

Variables	Total cohort				PSM cohort			
	Peribiliary group (*n* = 109)	Non-peribiliary group (*n* = 173)	*p*-value	SMD	Peribiliary group (*n* = 109)	Non-peribiliary group (*n* = 109)	*p*-value	SMD
Age at enrollment (years)*	58 (51–67)	59 (52–65)	0.935	0.05	58 (51–67)	58 (52–64)	0.514	0.12
No. of men	88 (80.7%)	126 (72.8%)	0.131	0.19	88 (80.7%)	90 (82.6%)	0.726	0.05
Etiology			0.531				0.811	
HBV	98 (89.9%)	150 (86.7%)			98 (89.9%)	94 (86.2%)		
HCV	2 (1.8%)	2 (1.2%)			2 (1.8%)	2 (1.8%)		
Others	9 (8.3%)	21 (12.1%)			9 (8.3%)	13 (11.9%)		
Antiviral treatment	94 (86.2%)	144 (83.2%)	0.499	0.23	94 (86.2%)	93 (85.3%)	0.846	0.03
AFP (U/L)*	9.2 (4.1–28.8)	8.7 (3.0–32.1)	0.742	0.08	9.2 (4.1–28.8)	10.0 (3.4–58.2)	0.574	0.107
ALT (U/L)*	33.0 (25.0–42.5)	30.0 (20.0–41.0)	0.059	0.04	33.0 (25.0–42.5)	31.0 (21.0–41.8)	0.255	0.096
AST (U/L)*	36.0 (27.5–48.5)	34.0 (26.0–48.0)	0.555	0.097	36.0 (27.5–48.5)	33.0 (26.0–44.0)	0.201	0.08
MELD score*	4.9 (2.9–7.1)	5.3 (3.1–7.4)	0.231	0.16	4.9 (2.9–7.1)	4.8 (2.9–6.7)	0.951	0.03
Child-Pugh class B	17 (15.6%)	45 (26.0%)	0.040	0.26	17 (15.6%)	16 (14.7%)	0.850	0.03
ALBI grade 2/3	69 (63.3%)	109 (63.0%)	0.960	0.01	69 (63.3%)	66 (60.6%)	0.676	0.06
Liver cirrhosis	94 (86.2%)	159 (91.9%)	0.127	0.18	94 (86.2%)	95 (87.2%)	0.842	0.03
Portal hypertension	82 (75.2%)	135 (78.0%)	0.586	0.07	82 (75.2%)	83 (76.1%)	0.875	0.02
Maximum tumor diameter (cm)*	2.5 (1.7–3.2)	2.0 (1.6–2.7)	0.002	0.41	2.5 (1.7–3.2)	2.4 (1.7–3.0)	0.424	0.13
Multiple tumors	30 (27.5%)	30 (17.3%)	0.042	0.25	30 (27.5%)	24 (22.0%)	0.347	0.13
TACE before RFA	53 (48.62%)	74 (42.77%)	0.336	0.117	53 (48.62%)	46 (42.20%)	0.341	0.128
Total ablation time (min)*	10 (8–13)	9.5 (6–12)	0.183	0.17	10.0 (8.0–13.0)	10.0 (7.0–13.0)	0.939	0.03
Tumor location (Couinaud segment)			0.207				0.333	
S1–4	24 (22.0%)	36 (20.8%)			24 (22.0%)	19 (17.4%)		
S5	17 (15.6%)	15 (8.7%)			17 (15.6%)	10 (9.2%)		
S6	21 (19.3%)	37 (21.4%)			21 (19.3%)	23 (21.1%)		
S7	15 (13.8%)	39 (22.5%)			15 (13.8%)	24 (22.0%)		
S8	32 (29.4%)	46 (26.6%)			32 (29.4%)	33 (30.3%)		

Unless otherwise noted, data are numbers of patients, with percentages in parentheses

*AFP* alpha-fetoprotein, *ALBI* albumin-bilirubin, *ALT* alanine aminotransferase, *AST* aspartate aminotransferase, *HBV* hepatitis B virus, *HCV* hepatitis C virus, *MELD* model for end-stage liver disease, *PSM* propensity score matching, *RFA* radiofrequency ablation, *SMD* standardized mean difference, *TACE* transarterial chemoembolization

* Data are medians, with interquartile ranges in parentheses

### LTP

**Fig. 3:**
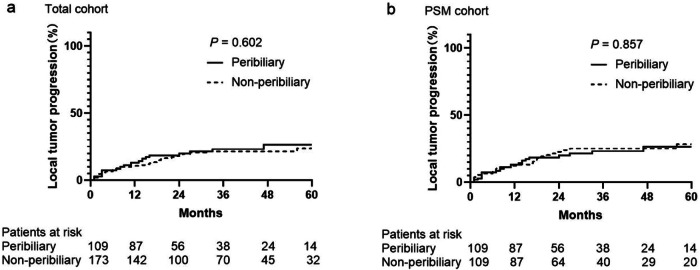
Cumulative LTP curves of the peribiliary and non-peribiliary groups. There was no significant difference in cumulative LTP between the peribiliary and non-peribiliary groups in the total cohort (**a**) and PSM cohort (**b**)

The median follow-up period was 40.0 (range, 35.6–44.4) months. In the total cohort (*n* = 282), 23 (21.1%) of 109 patients in the peribiliary group and 33 (19.1%) of 173 patients in the non-peribiliary group experienced LTP. Before PSM, there were no significant differences in the 1-, 3-, and 5-year LTP rates between peribiliary and non-peribiliary groups (13.0%, 23.1%, and 26.3% vs. 10.6%, 21.4%, and 23.6%, respectively, *p* = 0.602) (Fig. 3a). After PSM (*n* = 218), LTP was detected in 23 (21.1%) of 109 patients in the peribiliary group and 25 (22.9%) of 109 in the non-peribiliary group. There were still no significant differences in the cumulative LTP rates at 1, 3, and 5 years between the peribiliary and non-peribiliary groups (13%, 23.1%, and 26.3% vs. 12.1%, 25.1%, and 28.2%, respectively, *p* = 0.857) (Fig. 3b).

### OS

**Fig. 4:**
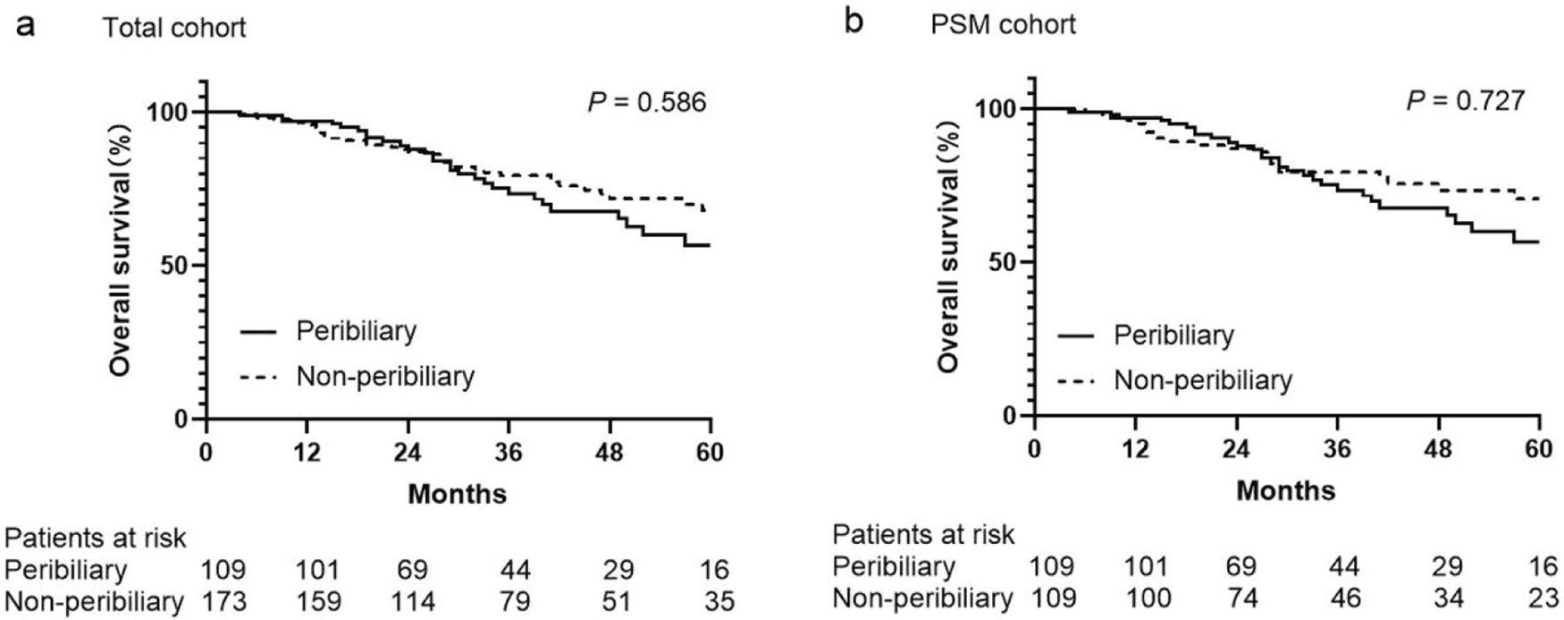
OS curves were of the peribiliary and non-peribiliary groups. There was no significant difference in OS between the peribiliary and non-peribiliary groups in the total cohort (**a**) and PSM cohort (**b**)

Among all patients, 29 (26.6%) of 109 in the peribiliary group and 44 (25.4%) of 173 in the non-peribiliary group had died. The estimated 1-, 3-, and 5-year OS rates of the peribiliary group were 97.2%, 73.5% and 56.6%, respectively, and 95.9%, 79.4% and 68.0% in the non-peribiliary group (*p* = 0.586) (Fig. 4a). After PSM, the estimated 1-, 3-, and 5-year OS rates in the peribiliary group were 97.2%, 73.5%, and 56.6%, respectively, and 95.3%, 79.5%, and 70.6% in the non-peribiliary group (*p* = 0.727) (Fig. 4b).

### DFS

**Fig. 5:**
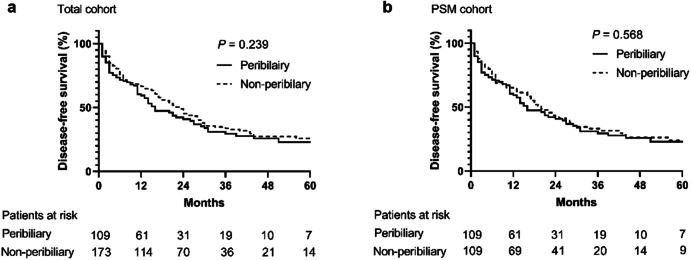
DFS curves of the peribiliary and non-peribiliary groups. There was no significant difference in DFS between the peribiliary and non-peribiliary groups in the total cohort (**a**) and PSM cohort (**b**)

Among all patients, 75 (68.8%) of 109 in the peribiliary group and 115 (66.5%) of 173 in the non-peribiliary group had died or experienced recurrence. The 1-, 3-, and 5-year DFS rates were 59.4%, 29.4%, and 22.9% in the peribiliary group, respectively, and 66.4%, 33.8%, and 25.7% in the non-peribiliary group (*p* = 0.239) (Fig. 5a). After PSM, the 1-, 3-, and 5-year DFS rates were 59.4%, 29.4%, and 22.9% in the peribiliary group, respectively and 64.2%, 33.1%, and 23.8% in the non-peribiliary group (*p* = 0.568) (Fig. 5b). Among patients who experienced recurrence, 26 (35.6%) of 73 in the peribiliary group and 46 (43.4%) of 106 in non-peribiliary group received curative treatment (ablation or surgical resection), with no significant difference (*p* = 0.353). None received liver transplantation for tumor recurrence after RFA in our study. Further details are provided in Supplementary Table 1.

### Prognostic factors for LTP and OS

Univariable analysis of the total cohort identified tumor diameter (*p* = 0.030) and tumor number (*p* = 0.031) as significant factors influencing LTP. The results of multivariable analysis revealed that AFP (hazard ratio (HR) = 2.60, 95% confidence interval (CI) = 1.09–6.19, *p* = 0.031), portal hypertension (HR = 2.27, 95% CI = 1.06–4.86, *p* = 0.035), tumor number (HR = 1.85, 95% CI = 1.05–3.24, *p* = 0.033), and tumor diameter (HR = 1.84, 95% CI = 1.01–3.36, *p* = 0.047) were independent prognostic factors for LTP. Univariate analysis identified other etiology (*p* = 0.019), MELD score (*p* = 0.008), tumor number (*p* = 0.010), and ablation time (*p* = 0.008) as significant factors influencing OS. Multivariate analysis confirmed other etiology (HR = 2.14, 95% CI = 1.13–4.03, *p* = 0.019), MELD score (HR = 1.96, 95% CI = 1.16–3.32, *p* = 0.013), tumor diameter (HR = 1.83, 95% CI = 1.09–3.10, *p* = 0.023), and tumor number (HR = 1.98, 95% CI = 1.18–3.32, *p* = 0.009) were independent risk factors for OS. Peribiliary location was not a prognostic factor for LTP (*p* = 0.622) or OS (*p* = 0.587) (Table 2).

**Table 2:** Risk factor analysis for LTP and OS in the total cohort

Variables	LTP				OS			
	Univariate analysis		Multivariate analysis		Univariate analysis		Multivariate analysis	
	HR (95% CI)	*p*-value	HR (95% CI)	*p*-value	HR (95% CI)	*p*-value	HR (95% CI)	*p*-value
Peribiliary vs non-peribiliary	1.14 (0.67–1.95)	0.622			1.14 (0.71–1.82)	0.587		
Age (> 65 years)	0.84 (0.44–1.58)	0.581			1.20 (0.71–2.01)	0.493		
Sex (female)	0.41 (0.19–0.91)	0.029	0.48 (0.22–1.08)	0.076	0.64 (0.36–1.15)	0.133		
Etiology (ref.: HBV)								
HCV	1.13 (0.16–8.19)	0.905			0.67 (0.09–4.81)	0.686	0.44 (0.06–3.24)	0.424
Others	1.58 (0.75–3.35)	0.231			2.11 (1.13–3.95)	0.019	2.14 (1.13–4.03)	0.019
Antiviral treatment	0.95 (0.47–1.95)	0.899			0.82 (0.46–1.47)	0.509		
AFP (> 200 ng/mL)	2.18 (0.93–5.10)	0.071	2.60 (1.09–6.19)	0.031	1.38 (0.59–3.19)	0.457		
MELD score (≥ 8)	1.77 (0.95–3.30)	0.071			2.03 (1.20–3.43)	0.008	1.96 (1.16–3.32)	0.013
Cirrhosis	1.69 (0.61–4.69)	0.309			1.76 (0.71–4.36)	0.224		
Portal hypertension	1.94 (0.92–4.09)	0.084	2.27 (1.06–4.86)	0.035	1.24 (0.69–2.22)	0.469		
Tumor diameter (≥ 2 cm)	1.92 (1.06–3.47)	0.03	1.84 (1.01–3.36)	0.047	1.66 (0.99–2.77)	0.054	1.83 (1.09–3.10)	0.023
Tumor number (≥ 2)	1.86 (1.06–3.25)	0.031	1.85 (1.05–3.24)	0.033	1.94 (1.17–3.22)	0.01	1.98 (1.18–3.32)	0.009
Tumor location (Couinaud segment, ref: S1–S4)								
S5	1.58 (0.63–3.93)	0.327			0.97 (0.38–2.49)	0.943		
S6	0.80 (0.32–1.99)	0.631			1.59 (0.82–3.08)	0.172		
S7	0.85 (0.34–2.11)	0.727			1.00 (0.47–2.12)	0.998		
S8	1.53 (0.74–3.17)	0.255			0.91 (0.47–1.78)	0.789		
TACE before RFA	1.00 (0.59–1.69)	1			1.18 (0.75–1.88)	0.474		
Total ablation time	1.04 (1.00–1.08)	0.082			1.05 (1.01–1.09)	0.008		

*AFP* alpha-fetoprotein, *ALBI* albumin-bilirubin, *ALT* alanine aminotransferase, *AST* aspartate aminotransferase, *CI* confidence interval, *HBV* hepatitis B virus, *HCC* hepatocellular carcinoma, *HCV* hepatitis C virus, *HR* hazard ratio, *LTP* local tumor progression, *MELD* model for end-stage liver disease, *OS* overall survival

### Subgroup analyses

**Fig. 6:**
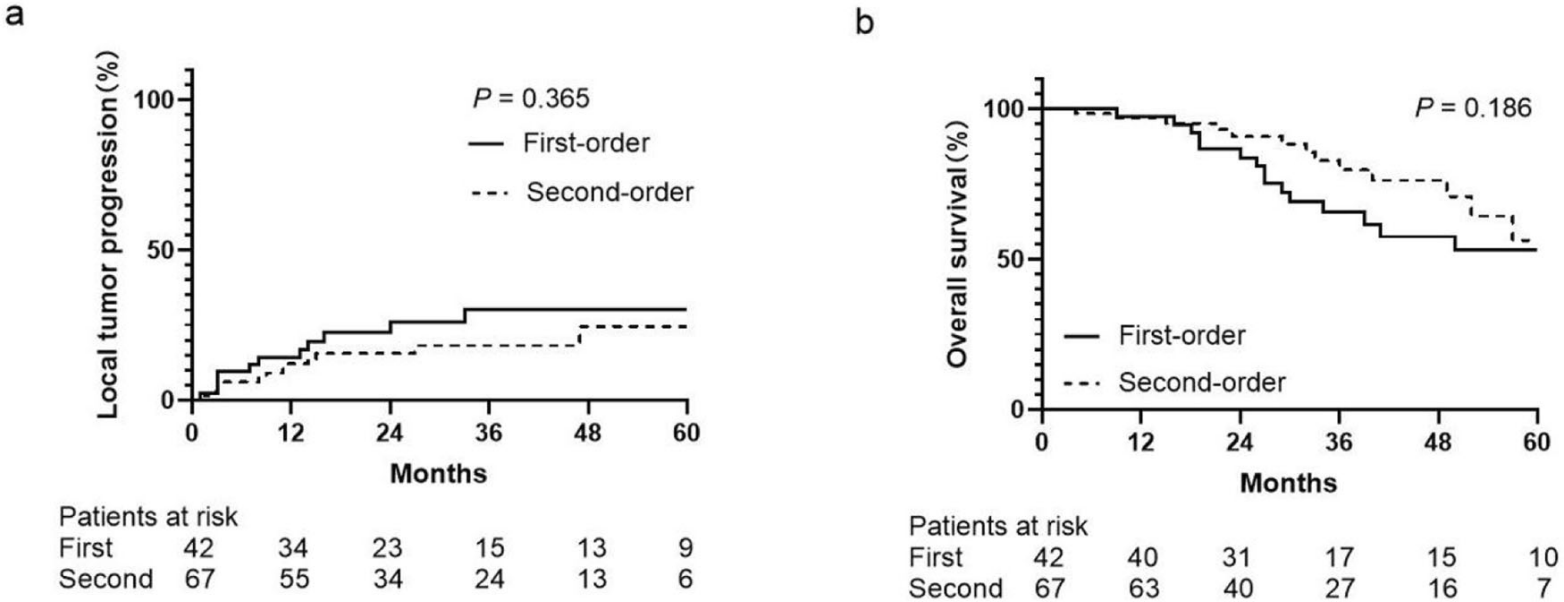
LTP and OS curves of the first- and second-order branch groups. There were no significant differences in (**a**) LTP and (**b**) OS between the two groups

The results of subgroup analyses based on maximum tumor diameter (≤ 2 cm or > 2 cm) and tumor number (1 vs. ≥ 2) are shown in Table 3. There were no significant differences in OS and LTP between subgroups in the total and PSM cohorts (all, *p* > 0.05). For peribiliary HCC, subgroup analysis of groups divided by first-order vs. second-order branches found no significant differences in LTP (*p* = 0.365) and OS (*p* = 0.186) (Fig. 6). There were no significant differences in OS and LTP between first-degree branches subgroup and non-peribiliary HCCs in the total and PSM cohorts (*p* = 0.213 to 0.684). No significant differences were also observed between second-degree branches subgroups and non-peribiliary HCCs for both cohorts (*p* = 0.542 to 0.950).

**Table 3:** Subgroup analyses according to maximum tumor diameter and tumor number

Variable	Mean overall survival time (month)			Mean local tumor progression (month)		
	Peribiliary	Non-peribiliary	*p*-value	Peribiliary	Non-peribiliary	*p*-value
Total cohort						
Tumor diameter						
< 2 cm	94.1 (79.4–108.7)	74.4 (64.2–84.6)	0.287	99.2 (89.7–108.7)	83.5 (75.0–89.7)	0.507
≥ 2 cm	65.4 (55.4–75.3)	67.9 (60.0–75.9)	0.443	72.7 (62.5–82.9)	73.7 (65.7–81.6)	0.584
Tumor number						
Single	79.1 (68.1–90.2)	74.4 (67.6–81.3)	0.764	82.8 (71.9–93.6)	84.6 (78.5–90.7)	0.143
2–3	48.2 (41.1–55.3)	58.0 (41.7–74.2)	0.655	52.7 (44.7–60.7)	57.6 (41.2–74.0)	0.075
Tumor location						
First-order branch	64.9 (52.0–77.7)	71.9 (65.4–78.4)	0.213	73.8 (60.6–87.0)	80.2 (74.2–86.3)	0.320
Second-order branch	77.9 (64.0–91.8)	71.9 (65.4–78.4)	0.777	86.4 (74.8–98.1)	80.2 (74.2–86.3)	0.950
PSM cohort						
Tumor diameter						
< 2 cm	94.1 (79.4–108.7)	70.7 (56.1–85.2)	0.173	99.2 (89.7–108.7)	80.4 (66.4–94.5)	0.459
≥ 2 cm	65.4 (55.4–75.3)	69.0 (60.2–77.8)	0.320	72.7 (62.5–82.9)	70.8 (61.6–80.1)	0.924
Tumor number						
Single	79.1 (68.1–90.2)	75.0 (66.5–83.5)	0.732	82.8 (71.9–93.6)	80.6 (72.2–89.1)	0.484
2–3	48.2 (41.1–55.3)	43.3 (34.2–52.4)	0.481	52.7 (44.7–60.7)	39.8 (28.5–51.1)	0.098
Tumor location						
First-order branch	64.9 (52.0–77.7)	70.8 (62.7–78.9)	0.296	73.8 (60.6–87.0)	76.4 (68.4–84.5)	0.684
Second-order branch	77.9 (64.0–91.8)	70.8 (62.7–78.9)	0.707	86.4 (74.8–98.1)	76.4 (68.4–84.5)	0.542

*PSM* propensity score matching

### Complications

There was no significant difference in the complication rate between the peribiliary and non-peribiliary groups in the total cohort (41.3% vs. 47.4%, respectively, *p* = 0.315) or the PSM cohort (41.3% vs. 45.0%, respectively, *p* = 0.584) (Table 4). Mild intrahepatic bile duct dilatation was more common in the peribiliary group than the non-peribiliary group (total cohort: 9.2% vs. 2.3%, respectively, *p* = 0.010; PSM cohort: 9.2% vs. 2.8%, respectively, *p* = 0.045). There were no significant differences in the minor complication rate, major complication rate, severe intrahepatic bile duct dilatation, biliary fistula, and biloma formation between the peribiliary and non-peribiliary groups in both cohorts (all, *p* > 0.05). No significant differences were observed for postoperative liver decompensation rates between the two groups for both cohorts (total cohort: 23.9% vs. 16.8%, *p* = 0.143; PSM cohort: 23.9% vs. 15.6%, *p* = 0.126). There were no treatment-related deaths in the two groups. Biloma formation (*n* = 1) and biliary fistula (*n* = 1) were treated with percutaneous biliary drainage. There was no significant difference in the minor and major complication rates between the non-TACE and TACE groups (32.90% vs. 39.37%, *p* = 0.260; 9.68% vs. 8.66%, *p* = 0.769, respectively) (Supplementary Table 2).

**Table 4:** Complications after radiofrequency ablation

	Total cohort (*n* = 282)			PSM cohort (*n* = 218)		
	Peribiliary group (*n* = 109)	Non-peribiliary group (*n* = 173)	*p*-value	Peribiliary group (*n* = 109)	Non-peribiliary group (*n* = 109)	*p-*value
Complication	45 (41.3%)	82 (47.4%)	0.315	45 (41.3%)	49 (45.0%)	0.584
Minor complication	33 (30.3%)	68 (39.3%)	0.123	33 (30.3%)	41 (37.6%)	0.253
Major complication	12 (11.0%)	14 (8.1%)	0.410	12 (11.0%)	9 (8.3%)	0.491
Intrahepatic bile duct dilatation	12 (11.0%)	6 (3.5%)	0.012	12 (11.0%)	4 (3.7%)	0.038
Mild intrahepatic bile duct dilatation	10 (9.2%)	4 (2.3%)	0.010	10 (9.2%)	3 (2.8%)	0.045
Severe intrahepatic bile duct dilatation	2 (1.8%)	2 (1.2%)	0.642	2 (1.8%)	1 (0.9%)	1.000
Biloma formation	1 (0.9%)	0 (0.0%)	0.387	1 (0.9%)	0 (0.0%)	0.387
Biliary fistula	1 (0.9%)	0 (0.0%)	0.387	1 (0.9%)	0 (0.0%)	0.387
Postoperative liver decompensation	26 (23.9%)	29 (16.8%)	0.143	26 (23.9%)	17 (15.6%)	0.126
Thrombosis in peritumoral vessel	6 (5.5%)	4 (2.3%)	0.192	6 (5.5%)	3 (2.8%)	0.499
Pain required treatment	6 (5.5%)	13 (7.5%)	0.512	6 (5.5%)	10 (9.2%)	0.299
Infection	5 (4.6%)	9 (5.2%)	0.817	5 (4.6%)	7 (6.4%)	0.553
Hydropneumothorax require drainage	1 (0.9%)	2 (1.2%)	1.000	1 (0.9%)	2 (1.8%)	1.000
Death	0 (0.0%)	0 (0.0%)	1.000	0 (0.0%)	0 (0.0%)	1.000

*PSM* propensity score matching

## Discussion

The challenging location of tumors may increase the difficulty of RFA and influence the prognosis of HCC patients. Recent advancements in RFA techniques have allowed for better control of tumors and improved patient prognosis. At present, there are no established guidelines for RFA of peribiliary HCC. The results of this study demonstrated that the long-term therapeutic outcomes of RFA for peribiliary HCC were similar to those for non-peribiliary HCC in terms of LTP, OS, and DFS before and after PSM. While mild intrahepatic bile duct dilatation after RFA for HCC was more frequent in the peribiliary group than the non-peribiliary group, these results suggest that RFA is suitable as an alternative method for treatment of peribiliary HCC.

Previous studies revealed that the presence of a peritumoral bile duct might be associated with poorer local tumor control and that RFA resulted in poorer local tumor control and a higher rate of recurrence of tumors in challenging locations, including perivascular, subcapsular, and periportal HCCs [47, 48]. In contrast, other studies supported the effectiveness of RFA for the treatment of HCC in challenging locations [22–28]. The results of the present study revealed no significant difference in LTP between peribiliary and non-peribiliary HCC before and after PSM, which was likely due to rapid improvements in RFA techniques in recent years, such as the introduction of multichannel RFA systems, fusion imaging guidance, and artificial fluid infusion [18, 49]. Multichannel systems are designed to use different electricity or activation modes to enhance the efficiency of ablation energy delivery [49]. Fusion imaging enables more accurate ablation of small invisible hepatic tumors, which are difficult to identify with conventional ultrasound guidance [50, 51]. Artificial fluid infusion can enhance the sonographic window and reduce the risks of thermal injury to adjacent abdominal organs and other complications [52, 53]. Besides, multiple electrodes and more powerful generators were recently introduced to supply a sufficient radiofrequency current to the liver parenchyma adjacent to the peritumoral bile duct to achieve complete tumor necrosis [54].

Recent advancements in ablation techniques have extended the application of thermal ablation to 3–5 cm HCC, and provide better control of the tumors, thereby improving prognosis [17–20]. Although RFA is the first-line treatment option for ≤ 3 cm HCC, in real life non-negligible part of RFAs are performed in 3–5 cm HCC [15, 16]. The disadvantages of RFA for treatment of HCC include incomplete ablation and damage to surrounding tissues. Complete tumor ablation may be compromised in tumors greater than 3 cm or in anatomically challenging locations (i.e., perivascular and subcapsular) [55]. In this study, survival analyses showed that tumor diameter was an independent prognostic factor for LTP and OS. Hence, future studies should explore new sensitive and predictive monitoring techniques for the RFA (i.e., hyperspectral imaging and real-time physics-based software) or other ablation modalities (i.e., cryotherapy and irreversible electroporation) [55–57].

Previous research has revealed that TACE combined with RFA is superior to RFA alone in inducing higher complete necrosis and improving patient prognosis, especially for > 3 cm HCC. TACE could increase the extent of the ablative necrosis zone by reducing the heat sink effect and inducing ischemia and inflammation in the treated tumor. Combined therapy has no advantage for small lesions < 3 cm since RFA can reach complete necrosis alone [12–14]. In our study, 82.3% (232/282) of HCCs included in our study were not greater than 3 cm. This may explain why TACE before RFA did not influence the results of LTP and OS. Another reason might be the recent advances in thermal ablation techniques, which enable larger and more efficient ablation [12, 15, 16]. Besides, there was no significant difference in the complication rates between the non-TACE and TACE groups, which was consistent with the previous studies [9–11].

There was no significant difference in OS between peribiliary and non-peribiliary HCC. Similar findings were reported in a previous study that found no significant difference in OS between the perivascular and non-perivascular groups after RFA for small HCC (≤ 3 cm) [58]. Severe intrahepatic bile duct dilatation after RFA might result in poorer survival outcomes due to impaired hepatic function caused by bile duct injury [8]. In this study, there was no significant difference in the incidence of severe bile duct dilatation between the peribiliary and the non-peribiliary groups. As the estimated 5-year recurrence rate of HCC was ~70% after initial therapy due to intrahepatic metastasis or de novo tumors, second-line treatment with RFA could be an effective strategy to improve OS of HCC patients [59–62]. Due to the absence of a control group, such as patients receiving alternative treatments or no treatment, it was challenging to assess the comparative effectiveness of RFA for peribiliary vs. non-peribiliary HCC. Hence, future studies should compare the long-term outcomes of different treatment modalities.

A peribiliary location of HCC was not a prognostic factor for OS after RFA in this study. A previous study also indicated that challenging subcapsular perivascular locations were not risk factors for OS [63, 64]. AFP, tumor diameter, and tumor number were identified as significant prognostic factors for HCC patients in this study, in agreement with recent reports [65–68]. The MELD score was a significant independent risk factor for OS in the current study, consistent with a previous study [69]. The MELD score is used to assess liver function, and hepatic dysfunction might influence the survival outcomes of HCC patients [70, 71]. The follow-up will be continued to update the data of this cohort and improve the reliability of long-term outcomes.

The results of the present study showed that mild intrahepatic bile duct dilatation, which required no additional treatment or hospitalization, occurred more frequently after PSM in the peribiliary group (10/109, 9.2%) than in the non-peribiliary group (3/109, 2.3%). Previous studies have indicated that peribiliary HCC patients might experience more major complications after RFA, such as severe bile duct dilatation [8, 10]. In contrast, there were no significant differences in the major complication rate and incidence of severe intrahepatic bile duct dilatation between the two groups in the present study, likely because of good control of the thermal field and precise electrode placement near the bile duct by experienced operators, in addition to thermal ablation monitoring to reduce the risk of major complications.

There were several limitations in this study. First, this retrospective study was inherently limited by the availability and quality of the data retrieved from medical records, which have introduced bias and confounding variables. The PSM method was used to balance the baseline characteristics between groups and minimize bias. Second, the inclusion criteria for this study were based on patients undergoing RFA from a single center, which may introduce selection bias and limit the generalizability of the findings to a broader HCC population. Thus, multicenter studies with larger cohorts are needed to validate the efficacy of RFA for the treatment of peribiliary HCC. Third, most of the patients in this study had hepatitis B virus infection; therefore, the results may not be applicable for other etiologies.

In conclusion, the long-term therapeutic outcomes of RFA for peribiliary HCC were like those for non-peribiliary HCC, suggesting that RFA presents a viable alternative for the treatment of peribiliary HCC.

## Supplementary information

ELECTRONIC SUPPLEMENTARY MATERIAL

## Data Availability

Data generated or analyzed during the study are available from the corresponding author by request.

## Abbreviations

AFP: Alpha fetoprotein
ALBI: Albumin-bilirubin
CT: Computer tomography
DFS: Disease-free survival
HCC: Hepatocellular carcinoma
HR: Hazard ratio
LTP: Local tumor progression
MELD: Model for end-stage liver disease
MRI: Magnetic resonance imaging
OS: Overall survival
PSM: Propensity score matching
RFA: Radiofrequency ablation
SMD: Standardized mean difference
TACE: Transcatheter arterial chemoembolization
