# Supplementary material for: Radiofrequency ablation for peribiliary hepatocellular carcinoma: propensity score matching analysis
Source: Insights Imaging. 2025 Feb 19;16:45. doi: 10.1186/s13244-025-01919-5 (PMC11839967; doi:10.1186/s13244-025-01919-5)
Supplement: Supplementary file 1 — ELECTRONIC SUPPLEMENTARY MATERIAL [file 13244_2025_1919_MOESM1_ESM.pdf]

Radiofrequency ablation for peribiliary hepatocellular carcinoma: propensity score matching analysis

ELECTRONIC SUPPLEMENTARY MATERIAL

Supplementary Table 1. Retreatment for the first recurrence of hepatocellular carcinoma after radiofrequency ablation
Abbreviations: PSM, propensity score matching; TACE, transcatheter arterial chemoembolization

	Total cohort			PSM cohort		
	Peribiliary group (n = 73)	Non-peribiliary group (n = 106)	*p*-value	Peribiliary group (n =73)	Non-peribiliary group (n = 71)	*p*-value
Curative therapies	26 (35.6%)	46 (43.4%)	0.353	26 (35.6%)	29 (40.8%)	0.607
Ablation or combined with TACE	23	39		23	24	
Surgical resection	3	7		3	5	
Noncurative therapies	47 (64.4%)	60 (56.6%)		47 (64.4%)	42 (59.2%)	
TACE	31	43		31	31	
Systemic and other therapies	16	17		16	11	

Supplementary Table 2. Complications after radiofrequency ablation between the patients treated with TACE and without TACE

	Total (n = 282)	Without TACE (n = 155)	With TACE (n = 127)	*p*-value
Complication	127 (45.04%)	66 (42.58%)	61 (48.03%)	0.360
Minor complication	101 (35.82%)	51 (32.90%)	50 (39.37%)	0.260
Major complication	26 (9.22%)	15 (9.68%)	11 (8.66%)	0.769
Intrahepatic bile duct dilatation	18 (6.38%)	12 (7.74%)	6 (4.72%)	0.302
Mild Intrahepatic Bile Duct Dilatation	14 (4.96%)	9 (5.81%)	5 (3.94%)	0.472
Severe Intrahepatic Bile Duct Dilatation	4 (1.42%)	3 (1.94%)	1 (0.79%)	0.760
Biloma formation	1 (0.35%)	1 (0.65%)	0 (0.00%)	1.000
Biliary fistula	1 (0.35%)	1 (0.65%)	0 (0.00%)	1.000
Postoperative liver decompensation	55 (19.50%)	30 (19.35%)	25 (19.69%)	0.944
Thrombosis in peritumoral vessel	10 (3.55%)	5 (3.23%)	5 (3.94%)	1.000
Pain required treatment	19 (6.74%)	9 (5.81%)	10 (7.87%)	0.491
Infection	14 (4.96%)	7 (4.52%)	7 (5.51%)	0.702
Hydropneumothorax require drainage	3 (1.06%)	2 (1.29%)	1 (0.79%)	1.000

Note: TACE, transcatheter arterial chemoembolization.
